# Deep-Learning-Based Image Denoising in Imaging of Urolithiasis: Assessment of Image Quality and Comparison to State-of-the-Art Iterative Reconstructions

**DOI:** 10.3390/diagnostics13172821

**Published:** 2023-08-31

**Authors:** Robert Terzis, Robert Peter Reimer, Christian Nelles, Erkan Celik, Liliana Caldeira, Axel Heidenreich, Enno Storz, David Maintz, David Zopfs, Nils Große Hokamp

**Affiliations:** 1 Institute for Diagnostic and Interventional Radiology, University Hospital Cologne, 50937 Cologne, Germanydavid.maintz@uk-koeln.de (D.M.); david.zopfs@uk-koeln.de (D.Z.);; 2 Department of Urology, Uro-Oncology, Robot-Assisted and Specialized Urologic Surger, University Hospital Cologne, 50937 Cologne, Germany

**Keywords:** deep learning, diagnostic imaging, image enhancement, kidney calculi, tomography, X-ray computed

## Abstract

This study aimed to compare the image quality and diagnostic accuracy of deep-learning-based image denoising reconstructions (DLIDs) to established iterative reconstructed algorithms in low-dose computed tomography (LDCT) of patients with suspected urolithiasis. LDCTs (CTDIvol, 2 mGy) of 76 patients (age: 40.3 ± 5.2 years, M/W: 51/25) with suspected urolithiasis were retrospectively included. Filtered-back projection (FBP), hybrid iterative and model-based iterative reconstruction (HIR/MBIR, respectively) were reconstructed. FBP images were processed using a Food and Drug Administration (FDA)-approved DLID. ROIs were placed in renal parenchyma, fat, muscle and urinary bladder. Signal- and contrast-to-noise ratios (SNR/CNR, respectively) were calculated. Two radiologists evaluated image quality on five-point Likert scales and urinary stones. The results showed a progressive decrease in image noise from FBP, HIR and DLID to MBIR with significant differences between each method (*p* < 0.05). SNR and CNR were comparable between MBIR and DLID, while it was significantly lower in HIR followed by FBP (e.g., SNR: 1.5 ± 0.3; 1.4 ± 0.4; 1.0 ± 0.3; 0.7 ± 0.2, *p* < 0.05). Subjective analysis confirmed best image quality in MBIR, followed by DLID and HIR, both being superior to FBP (*p* < 0.05). Diagnostic accuracy for urinary stone detection was best using MBIR (0.94), lowest using FBP (0.84) and comparable between DLID (0.90) and HIR (0.90). Stone size measurements were consistent between all reconstructions and showed excellent correlation (r^2^ = 0.958–0.975). In conclusion, MBIR yielded the highest image quality and diagnostic accuracy, with DLID producing better results than HIR and FBP in image quality and matching HIR in diagnostic precision.

## 1. Introduction

Urolithiasis represents an increasing issue in industrialized countries [1]. Especially in younger patients, its incidence and reoccurrence are on the rise, which is primarily due to changes in consumption behavior and decreased physical activity [2].

In symptomatic patients with suspected urolithiasis, unenhanced computed tomography (CT) of the abdomen is the modality of choice due to its high diagnostic accuracy, high accuracy of size measurements and precise assessment of stone localization, which is relevant for treatment decision making [3,4]. Due to an increasing use of CT scans, especially in patients undergoing repetitive scanning, the radiation dose has to be as low as reasonably achievable [5]. As a consequence, low-dose CT protocols have been extensively investigated and are recommended by current guidelines in non-obese patients [6].

One of the main technical advances in CT imaging was the implementation of iterative reconstruction algorithms into clinical routine, which gradually replaced the standard of the past decades, filtered-back projection (FBP) [3,7]. As of today, hybrid and model-based iterative reconstruction algorithms (HIR and MBIR) are used across a wide range of clinical scenarios. HIR executes multiple iterations to reduce image noise and improve the signal-to-noise ratio of CT data; while the same applies to MBIR, here knowledge on the geometry of the acquisition process is added to the statistical model of noise [8]. Both have shown to significantly improve the image quality and hence the diagnostic accuracy of urinary stone detection, particularly when using low-dose techniques [3,9]. Despite these promising results, iterative reconstruction algorithms rely on powerful hardware and time-intensive analytical computations [10]. 

A more recent and thriving approach is the use of artificial intelligence in terms of deep-learning-based image reconstruction techniques (DLRs) [8]. So far, DLRs in CT have demonstrated promising results in different areas of use such as low-dose thoracic, abdominal and whole-body CT [11,12,13]. One of the commercially available DLRs performs deep-learning-based denoising of image data, resulting in a noise-reduced image (DLID, PixelShine, AlgoMedica) [14]. The fact that no raw CT data are needed and that hardware requirements are less demanding compared to iterative reconstruction suggests a large potential for widespread clinical use [15]. Yet, information about the efficacy of these deep-learning-based reconstructions for image denoising of low-dose CT scans is sparse. To our best knowledge, prior research has not conducted an in-depth assessment of subjective image quality and diagnostic precision regarding detectability of urinary stones compared to each common reconstruction technique, including the cutting-edge MBIR [14,16,17,18]. Furthermore, the impact of denoising in the removal of small structures (e.g., urinary stones) is unknown [14].

Hence, this study aimed to compare (i) image quality and (ii) diagnostic accuracy of (A) deep-learning-based image denoising compared to (B) state-of-the-art hybrid and (C) model-based iterative reconstruction algorithms in unenhanced, low-dose CT scans in suspected urolithiasis.

## 2. Materials and Methods

This retrospective, single-center study was performed after the institutional review board waived informed consent. A structured search in the picture archiving and communication system as well as the radiology information system was performed with the following inclusion criteria: (1) age ≥ 18 years, (2) unenhanced CT scan of the genitourinary system in patients with suspected urinary stones between April 2016 and June 2017, (3) standardized imaging protocol using a low-dose imaging technique with a volumetric computed tomography dose index (CTDI_vol_) of 2 mGy and (4) FBP, HIR and MBIR image reconstructions available for analysis. Out of these patients, 13 patients were excluded due to incomplete imaging data and 1 patient due to a genitourinary system filled with contrast media following a prior intervention. Eventually, 76 patients were identified eligible for this study.

### 2.1. Acquisition Parameters

All examinations were performed in the cranio-caudal direction and a head-first, supine position for clinical indications on the same 256-row computed tomography scanner (iCT, Philips Healthcare, Best, The Netherlands). Scan parameters were as follows: collimation 128 × 0.625 mm, matrix 512 × 512, tube voltage 100 kVp, fixed tube current-time product 50 mAs, pitch factor 0.977 and rotation time 0.5.

Images were reconstructed using a filtered-back projection reconstruction algorithm (FBP, Philips healthcare), a hybrid iterative reconstruction algorithm (HIR, iDose; Philips Healthcare) and a model-based iterative reconstruction algorithm (MBIR, IMR; Philips Healthcare).

Filtered back projection is a method used to correct blurring in analytic reconstruction. It works by using a convolution filter and calculating the attenuation coefficients in a cross-section based on ray sums at varying sine wave angles. This complex process involves an algorithm with 250,000 equations, which requires a high-powered computer to solve. Once solved, the computer stores a projection that represents the anatomy. Each pixel of this projection corresponds to a voxel of the actual image [8]. HIR combines traditional filtered-back projection with iterative methods, enhancing image clarity while reducing noise [7,8]. On the other hand, MBIR utilizes sophisticated models of the CT scanner’s physics and statistics to iteratively refine images, offering potential for significant dose reductions and superior noise suppression [8].

Throughout all reconstructions, a soft tissue kernel and medium denoising level has been used:

- FBP: soft tissue kernel (B, Philips Healthcare), no denoising.
- HIR: soft tissue kernel (B, Philips Healthcare) and medium denoising (3/7).
- MBIR: soft tissue kernel (Sharp Plus, Philips Healthcare) and medium denoising (2/3).

All images were reconstructed using a slice thickness of 2 mm and a section increment of 1 mm using a 512 × 512 matrix. Subsequently, FBP images were post-processed using a commercially available deep-learning-based image denoising algorithm (PixelShine, AlgoMedica, Sunnyvale, California, USA) with default presets. DLID, a component of artificial intelligence and machine learning, efficiently processes CT scans, consistently eliminates ‘noise’ and yields high-contrast images with intricate detail. Furthermore, DLID maintains complete image integrity, ensuring no data are omitted from the dataset [19].

The dose-length product and CTDI_vol_ were recorded from the radiation dose report. Size-specific dose estimate (SSDE) was determined based upon the effective diameter of patients and its corresponding conversion factor for the 32 cm phantom for CTDI_vol_ as suggested earlier [20]. The effective diameter was obtained by performing manual measurements of the abdominal anterior–posterior and lateral dimensions of each patient.

### 2.2. Quantitative Image Analysis

**Figure 1:**
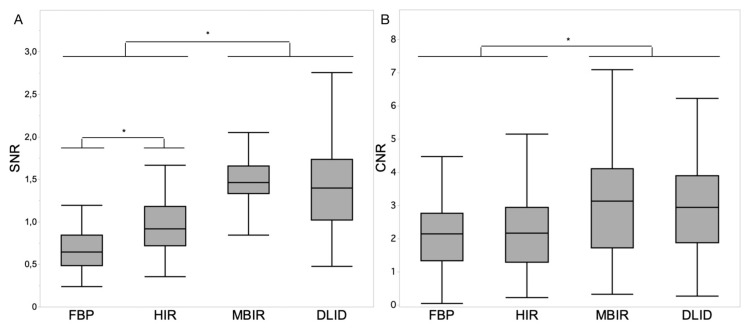
Boxplot diagrams depicting signal-to-noise ratio (SNR) of renal parenchyma (**A**) and contrast-to-noise ratio (CNR) of renal parenchyma vs. urine (**B**) within the different image reconstruction techniques. Asterisks indicate statistically significant differences (*p* < 0.05, respectively). Decimal signs are stated as commas.

Region of interest (ROI)-based quantitative image analysis was performed by a radiologist with two years of experience in abdominal imaging using the local picture archiving and communication system (Impax EE, Dedalus) to obtain the mean CT numbers in Hounsfield units (HU) and their standard deviation (SD). ROIs with a constant size (100 mm^2^) were placed in the following areas on MBIR and copied to identical positions on FBP, HIR and DLR: (1) upper and lower renal parenchyma of the left and right kidney, respectively, (2) subcutaneous fat of the anterior abdominal wall (n = 2), (3) psoas muscle on each side and (4) urine within the bladder (Figure 1).

The SD of muscle was defined as image noise, the signal-to-noise ratio (SNR) of renal parenchyma as SNR = HU/SD and the contrast-to-noise ratio (CNR) of the renal parenchyma vs. urine as the difference of the average Hounsfield units divided by the square root of the sum of the SD of the two ROIs.

### 2.3. Qualitative Image Analysis

Reference standard regarding the presence, number, location and size of urinary stones was assessed by one board-certified radiologist with six years of experience in abdominal imaging on MBIR images as it showed superior image quality and urinary stone detection as well as size assessment over FBP and HIR [21,22,23]. The location of urinary stones was classified as (1) pelvicalyceal system, (2) proximal ureter, (3) mid ureter, (4) distal ureter and (5) bladder for the right or left side, respectively.

Two radiologists with five years of experience in abdominal imaging independently evaluated image quality and noted the presence of urinary stones on all reconstructions in a randomized order. Readers were blinded to the reconstruction technique and were free to adjust window settings. Image quality was rated on 5-point Likert scales regarding image noise, overall image quality and delineation of the ureter (Table 1). Urinary stones were evaluated regarding presence, number, location and size. The maximum size of each stone was measured on multiplanar reformations (MPR) as suggested earlier [24]. All stones were annotated to compare findings with the reference standard regarding diagnostic accuracy and size of missed stones.

### 2.4. Statistical Analysis

Data processing and statistical analyses were performed using Microsoft Excel (Version 16.63.1, Microsoft Corporation, Redmond, Washington, DC, USA) and JMP Software (16.1.0), respectively, unless specified below. A *p*-value ≤ 0.05 was considered statistically significant.

The Shapiro–Wilk test was used to rule out normal distribution of the data. Steel–Dwass test was performed to compare non-parametric statistical data between the different reconstruction algorithms. For the detection rate of urinary stones, pooled sensitivity and specificity were calculated using a contingency table. Correlation of the urinary stone size measurements between the different reconstruction techniques was assessed using the coefficient of determination. Inter-rater reliability regarding the qualitative image analysis was determined by means of intra-class correlation estimates (intra-class correlation coefficient (ICC)) using R Studio (Version 2021.09.01, RStudio Inc., Boston, Massachusetts, USA). ICCs were calculated based on a mean of two raters, consistency and a 2-way mixed-effects model [25]. It was evaluated as described earlier: excellent (ICC > 0.8), good (ICC > 0.6), moderate (ICC > 0.4) and poor agreement (ICC < 0.4), [26].

## 3. Results

### 3.1. Study Characteristics

The study cohort comprised 76 patients with a mean age of 40.3 ± 15.2 years (range, 18–81 years). Out of these patients, 25 (32.9%) were women and 51 (67.1%) men. The dose-length product was 97.9 ± 13.2 mGy*cm, and SSDE was 2.7 ± 0.4 mGy.

In total, 104 stones were present within the urinary tract: 69 (66.3%) in the pelvicalyceal system, 17 (16.3%) in the upper ureter, 1 (1.0%) in the middle ureter, 12 (11.5%) in the distal ureter and 5 (4.8%) in the bladder. Out of the 99 stones in the pelvicalyceal system or ureter, 61 (61.6%) were on the right side and 38 (38.4%) on the left side.

### 3.2. Quantitative Image Analysis

Values of the quantitative image analysis are reported in Table 2.

#### 3.2.1. Attenuation

Attenuation in renal parenchyma, fat and urine were comparable between the different image reconstruction techniques (*p* > 0.05). Attenuation in muscle was significantly lower in MBIR compared to FBP, HIR and DLID, respectively (54.2 ± 4.1 HU vs. 58.1 ± 6.8 HU, 56.6 ± 4.8 HU and 58.0 ± 6.6 HU; *p* < 0.05).

#### 3.2.2. Noise

Image noise as defined by SD in muscle gradually decreased from FBP, HIR and DLID to MBIR with significant differences between each image reconstruction technique (71.3 ± 29.4, 44.7 ± 12.9, 33.3 ± 12.8 and 22.0 ± 5.6; each *p* < 0.05).

#### 3.2.3. Signal-to-Noise Ratio (SNR)

The SNR of renal parenchyma was comparable between MBIR and DLID (1.5 ± 0.3 and 1.4 ± 0.4; *p* > 0.05), while it was significantly lower in HIR followed by FBP (*p* < 0.05) (Figure 1A).

#### 3.2.4. Contrast-to-Noise Ratio (CNR)

The CNR for renal parenchyma vs. urine was significantly higher in MBIR and DLID compared to HIR and FBP (3.1 ± 1.7 and 3.0 ± 1.4 vs. 2.3 ± 1.2 and 2.2 ± 1.0; *p* < 0.05) (Figure 1B).

### 3.3. Qualitative Image Analysis

#### Image Quality

The overall intraclass correlation between the two independent readers was 0.792 (95% confidence interval, 0.763–0.818), indicating a good inter-reader reliability; here, no differences between reconstruction techniques were found.

**Figure 2:**
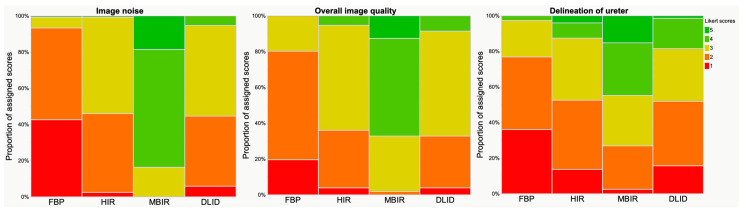
Results of qualitative analysis. Visualization with Likert scale (scoring between 1 and 5, see also Table 1). Image noise, overall image quality and delineation of ureter received significantly higher ratings in MBIR, followed by HIR/DLID and FBP.

Subjective image quality parameters were rated comparable between HIR and DLID (*p* > 0.05), while FBP received significantly lower and MBIR significantly higher scores with regards to all parameters: image noise, overall image quality and delineation of ureters, respectively (*p* < 0.05) (Figure 2). Qualitative results of subjective image parameters are reported in Table 3.

### 3.4. Urinary Stones

#### 3.4.1. Detection Rate

The diagnostic accuracy for urinary stone detection was best using MBIR (0.94, sensitivity 0.94, specificity 0.95), comparable between DLID (0.90, sensitivity 0.88, specificity 0.93) and HIR (0.90, sensitivity 0.89, specificity 0.91) and lowest using FBP (0.84, sensitivity 0.82, specificity 0.89).

#### 3.4.2. Stone Size Measurements

Of the 104 present urinary stones, 77 stones were detected on all image reconstruction techniques by both readers and showed an overall CT-based size of 7.2 ± 4.4 mm. Stone size measurements were comparable between the different image reconstruction techniques (FBP, 7.3 ± 4.3 mm; HIR, 7.2 ± 4.5 mm; MBIR, 7.3 ± 4.4 mm; DLID, 7.1 ± 4.6 mm; *p* > 0.05) and showed good correlation (r^2^ = 0.958–0.975). The intraclass correlation between the two independent readers was 0.983 (95% confidence interval, 0.979–0.987), indicating an excellent inter-reader reliability. The size of missed urinary stones based on the reference standard was 3.5 ± 1.5 mm and comparable between the different image reconstruction techniques (FBP, 3.3 ± 1.5 mm; HIR, 3.9 ± 1.4 mm; MBIR, 3.2 ± 1.7 mm; DLID, 3.6 ± 1.5 mm; *p* > 0.05).

## 4. Discussion

**Figure 3:**
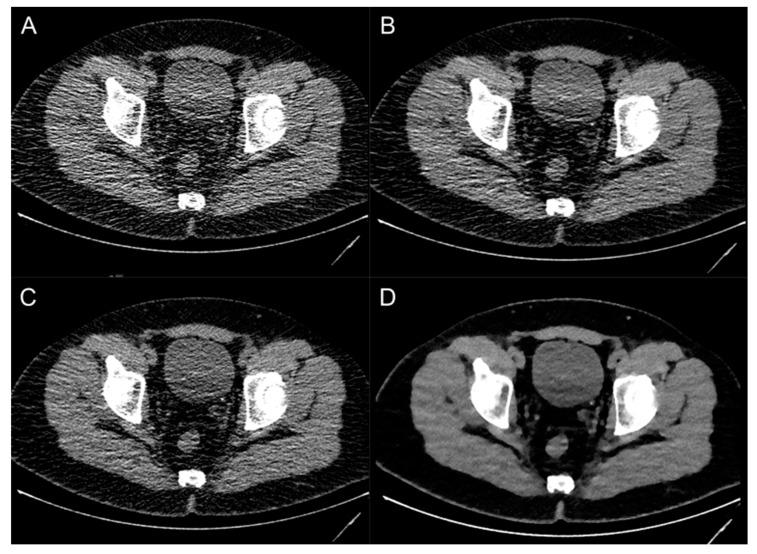
Image examples of a patient with an ostial ureteral stone on the left side using different image reconstruction techniques: filtered-back projection (**A**), deep-learning-based image denoising (**B**), hybrid iterative reconstruction (**C**) and model-based iterative reconstruction (**D**).

**Figure 4:**
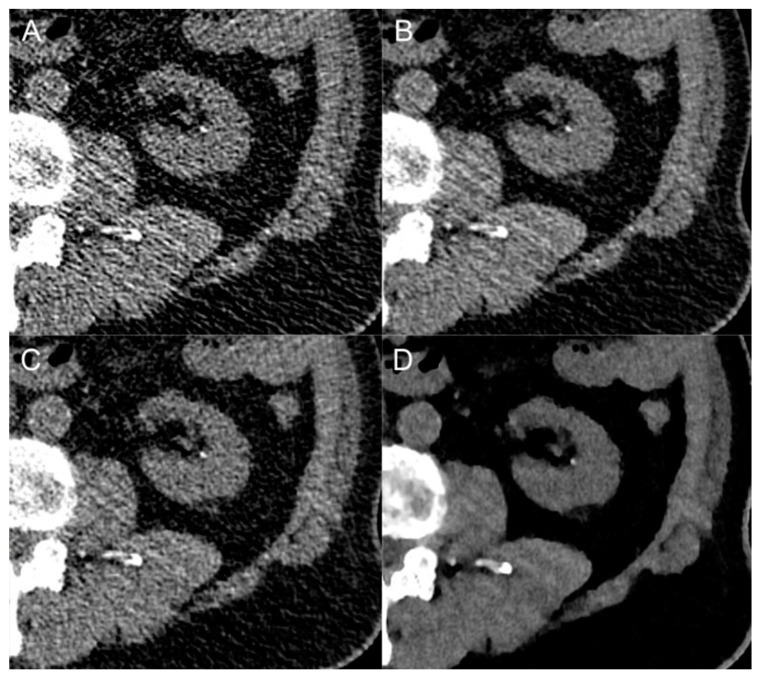
Close-up comparison of a kidney stone in the left renal calyx in different image reconstruction techniques: filtered-back projection (**A**), deep-learning-based image denoising (**B**), hybrid iterative reconstruction (**C**) and model-based iterative reconstruction (**D**).

This retrospective study investigated the image quality of a deep-learning-based image denoising reconstruction technique for the evaluation of unenhanced, low-dose CT scans in suspected urolithiasis compared to state-of-the-art hybrid and model-based iterative reconstruction algorithms. DLID images showed significantly lower image noise and improved S-/CNR compared to HIR and FBP. Subjective image quality and diagnostic accuracy of urinary stone detection was similar to HIR and better compared to FBP. While SNR and CNR were comparable with MBIR, image noise, subjective image quality and diagnostic accuracy of urinary stone detection was best in MBIR (Figure 3 and Figure 4). Size measurements of urinary stones were independent of the image reconstruction technique.

Deep-learning-based reconstruction algorithms have already been investigated in other clinical areas, e.g., one study group evaluated the value of DLID in ultra-low-dose pediatric thorax CT, low-dose whole-body CT and peri-interventional cone-beam CT of the liver [12,13,27]. These studies report on improvement of image quality compared to FBP and HIR, e.g., yielding a reduction of radiation dose of up to 30% in whole-body CT regardless of the scanner type [12,13]. However, data about the effect of DLID on the evaluation of unenhanced low-dose CT in patients with suspected urinary stone formation are scarce (Table 4).

The results of the few earlier studies investigating DLID and other DLR for the evaluation of low-dose CT scans in suspected urinary stones are mainly in line with the results of our study. While Delabie et al., Thapaliya et al. and Zhang et al. investigated vendor-specific commercially available deep-learning-based reconstruction algorithms, to the best of our knowledge, only Steuwe et al. also investigated DLID for the assessment of urinary stones [14,16,17,18].

Steuwe et al. evaluated objective image quality, the size as well as CT attenuation values of urinary stones in DLID-processed images compared to FBP and HIR in low-dose CT scans of 45 patients with urinary stones. All urinary stones were detected in the three different image reconstructions, while size measurements and attenuation values were comparable. In line with our study, DLID significantly reduced image noise and as a consequence improved SNR and CNR. Contrary to the study by Steuwe et al., we included a detailed subjective image quality evaluation and the detectability of urinary stones [14].

In another approach, Delabie et al. compared a different, vendor-specific DLR (TrueFidelity, GE Healthcare, Chicago, IL, USA) with FBP and HIR regarding image quality, CT attenuation values and detectability of urinary stones in 75 patients with suspected urolithiasis undergoing low-dose CT. All image quality parameters were significantly raised, and detectability was excellent for stones > 3 mm in DLR images, respectively. Contrary to the studies of Steuwe et al. and Zhang et al., which both reported comparable attenuation values between DLR and HIR, Delabie et al. reported lower attenuation values of urinary stones when using deep-learning-based reconstruction techniques [16].

Furthermore, Thapaliya et al. compared another vendor-specific DLR (Advanced intelligent Clear-IQ Engine (AiCE), Canon Medical Systems) with different degrees of noise reduction and sharpening available to HIR in 14 unenhanced CT scans. Results showed similar stone detectability and stone size measurements between all different DLR used compared to HIR, whereas image quality was not assessed [17]. The same vendor-specific DLR (AiCE) was evaluated by Zhang et al. in a prospective study regarding diagnostic accuracy and image quality of ultra-low-dose CT in 60 patients with suspected urinary stones. Objective image quality was higher in ultra-low-dose CT with DLR compared to HIR, irrespective of the used radiation dose, which is partly in line with the results of our study. Furthermore, detectability of urinary stones was similar between ultra-low-dose CT using DLR compared to HIR, while stone size and attenuation values were comparable between all the different image reconstruction techniques. In addition, the average size of missed stones was slightly smaller (<3 mm) compared to our study (3.5 ± 1.5 mm), yet there was no significant difference in stone size between the evaluated image reconstruction techniques in our study [18].

The aforementioned studies and the results of our study have in common that image noise was decreased in DLR, which lead to an increase in SNR and CNR as well as subjective image quality when comparing DLR with FBP and HIR. Furthermore, diagnostic accuracy and stone size measurements seem to remain unaffected by DLR, whereas attenuation values of urinary stones may vary [14,16,17,18]. In the study by Zhang et al., these findings showed the potential of DLR to reduce radiation dose by 76.6%, while maintaining superior image quality and comparable diagnostic accuracy compared to HIR [18]. Yet, all of the published studies only compared different DLR techniques with FBP and HIR, although MBIR images have shown superior image quality and urinary stone detection [22,28]. Hence, studies addressing a direct comparison between the different DLR techniques and MBIR are encouraged in order to further evaluate their diagnostic benefit.

Several technical differences exist between the commercially available DLR techniques. Whilst the DLID investigated in this study is compatible with all CT scanners from different vendors and only needs FBP image data for image reconstruction, making it more widely accessible, most other DLR algorithms are bound to vendor-specific CT scanners and need raw data for image processing [19,29,30]. Additionally, DLID software is a flexible system and parameters can be adjusted to the user’s preferences allowing for potentially optimized results adapted to radiologists’ needs regarding different examination protocols.

Apart from the retrospective study design, several limitations need to be discussed. First, this study included a limited number of patients and stones, respectively. Second, the reference standard regarding the presence of urinary stones was determined on MBIR images, which possibly affected the results of diagnostic accuracy in favor of MBIR. However, MBIR images have been chosen as the reference standard based on their superior image quality and urinary stone detection compared to FBP and HIR [22,28]. Opposed to MBIR, noise texture remains unaltered in DLID images, which have shown to comprise the least noise power spectrum shift [31], which is possibly preferred by some readers [32]. Lastly, we only included data from a single CT scanner, and therefore, results might vary between different vendors, scanners and protocols, respectively.

In conclusion, deep-learning-based image denoising reconstruction techniques increase the image quality of low-dose CT scans in patients with suspected urinary stones compared to FPB and HIR, while diagnostic accuracy is comparable to HIR and superior to FBP. MBIR remains the state-of-the-art reconstruction technique in the CT imaging of urinary stones, exhibiting the best image quality compared to all other reconstruction techniques.

## Figures and Tables

**Table 1:** Likert scales of qualitative analysis.

Likert Scale	Image Noise	Overall Image Quality	Delineation of Ureter
1	Excessive	Non-diagnostic	Poor
2	Severe	Suboptimal	Difficult
3	Moderate	Acceptable	Intermediate
4	Some	Good	Good
5	No visually perceptible noise	Excellent	Excellent

**Table 2:** Quantitative results of attenuation and noise in different tissues, SNR and CNR.

	FBP	HIR	MBIR	DLID
Attenuation				
Renal parenchyma	36.4 ± 5.0	35.8 ± 4.6	35.2 ± 4.6	36.5 ± 4.7
Fat	−119.2 ± 10.4	−118.5 ± 10.2	−119.5 ± 9.7	−117.9 ± 10.2
Muscle	58.1 ± 6.8	56.6 ± 4.8	54.2 ± 4.1	58.0 ± 6.6
Urine	13.5 ± 15.4	16.0 ± 12.9	15.0 ± 13.2	14.9 ± 14.5
Noise				
Renal parenchyma	62.1 ± 25.6	40.9 ± 12.1	24.3 ± 5.4	29.2 ± 12.0
Fat	34.0 ± 10.8	25.0 ± 6.7	18.5 ± 3.5	16.5 ± 4.8
Muscle	71.3 ± 29.4	44.7 ± 12.9	22.0 ± 5.6	33.3 ± 12.8
Urine	78.4 ± 30.3	44.9 ± 11.7	23.5 ± 5.1	35.1 ± 14.0
SNR	0.7 ± 0.2	1.0 ± 0.3	1.5 ± 0.3	1.4 ± 0.4
CNR	2.2 ± 1.0	2.3 ± 1.2	3.1 ± 1.7	3.0 ± 1.4

Results are means ± SDs. FBP, filtered-back projection; HIR, hybrid iterative reconstruction algorithm; MBIR, model-based iterative reconstruction algorithm; DLID, deep-learning-based image denoising reconstruction; SNR, signal-to-noise ratio; CNR, contrast-to-noise ratio.

**Table 3:** Qualitative results of subjective image parameters ^a^.

	FBP	HIR	MBIR	DLID
Image noise	2 (1–2)	3 (2–3)	4 (4–5)	3 (2–3)
Overall image quality	2 (2–3)	3 (3–3)	4 (4–4)	3 (3–3)
Delineation of ureters	2 (2–3)	3 (2–3)	4 (3–4)	3 (2–3)

^a^ Results are medians (quartiles). FBP, filtered-back projection; HIR, hybrid iterative reconstruction algorithm; MBIR, model-based iterative reconstruction algorithm; DLID, deep-learning-based image denoising reconstruction.

**Table 4:** Overview of studies on urinary stones investigation DLR.

	Zhang et al. [18]	Thapalia et al. [17]	Delabie et al. [16]	Steuwe et al. [14]	This Study
Deep-learning algorithm (commercial name), vendor	AiCE, Canon	AiCE, Canon	TrueFidelity, GE	PixelShine, AlgoMedica	PixelShine, AlgoMedica
Preprocessing data	Raw data	Raw data	Raw data	FBP	FBP
Techniques compared	DLR, HIR	DLR, HIR	DLR, HIR, FBP	DLID, HIR, FBP	DLID, MBIR, HIR, FBP
Type of CT scans included	Abdomen + pelvis, low-dose	Abdomen + pelvis, low-dose	Abdomen + pelvis, low-dose	Abdomen + pelvis, low-dose	Abdomen + pelvis, low-dose
N of patients	51	14	75	45	76
Objective Criteria	Noise, SNR, radiation dose	n/a	Attenuation, noise, S-/CNR	Attenuation, noise, S-/CNR	Attenuation, noise, S-/CNR
Subjective Criteria	Image quality	n/a	Image quality	n/a	Image quality, image noise, delineation of ureters
Task-based criteria	Stone detectability	Stone detectability, stone size measurement	Stone detectability	Stone size measurements	Stone detectability, stone size measurement
Conclusion	Comparable detectability and size assessment of urinary stones while reducing radiation dose.	Comparable detectability of urinary stones between DLR and HIR.	DLR image quality superior to HIR and FBP. Excellent detection of urinary stones.	DLID to decrease image noise significantly. Improved objective image quality while maintaining correct stone size measurements.	DLID produced better image quality than HIR and FBP and matching HIR in diagnostic precision. MBIR yielded the best results.

FBP, filtered-back projection; HIR, hybrid iterative reconstruction algorithm; DLR, deep-learning-based image reconstruction techniques; DLID, deep-learning-based image denoising reconstruction; SNR, signal-to-noise ratio; CNR, contrast-to-noise ratio.

## Data Availability

All data are available within the article.
